# Heme Oxygenase-1 and Brain Oxysterols Metabolism Are Linked to Egr-1 Expression in Aged Mice Cortex, but Not in Hippocampus

**DOI:** 10.3389/fnagi.2018.00363

**Published:** 2018-11-06

**Authors:** Paolo Rosa, Chiara Zerbinati, Alessio Crestini, Anna-Maria Canudas, Giuseppe Ragona, Annamaria Confaloni, Luigi Iuliano, Antonella Calogero

**Affiliations:** ^1^Department of Medical-Surgical Sciences and Biotechnologies, Sapienza University of Rome, Polo Pontino, Latina, Italy; ^2^Istituto Chirurgico Ortopedico Traumatologico, ICOT, Latina, Italy; ^3^Department of Neuroscience, Istituto Superiore di Sanità, Rome, Italy; ^4^Department of Pharmacology, Toxicology and Therapeutic Chemistry (Pharmacology Section), Institute of Neuroscience, CIBERNED, University of Barcelona, Barcelona, Spain

**Keywords:** Egr-1, aging brain, oxysterols, HO-1, oxidative stress, cortex, hippocampus

## Abstract

Throughout life, stress stimuli act upon the brain leading to morphological and functional changes in advanced age, when it is likely to develop neurodegenerative disorders. There is an increasing need to unveil the molecular mechanisms underlying aging, in a world where populations are getting older. Egr-1 (early growth response 1), a transcriptional factor involved in cell survival, proliferation and differentiation – with a role also in memory, cognition and synaptic plasticity, can be implicated in the molecular mechanism of the aging process. Moreover, Heme Oxygenase-1a (HO), a 32 kDa heat-shock protein that converts heme to iron, carbon monoxide and biliverdin, is a key enzyme with neuroprotective properties. Several *in vitro* and *in vivo* studies reported that HO-1 could regulate the metabolism of oxysterols, oxidation products of cholesterol that include markers of oxidative stress. Recently, a link between Egr-1 and HO-1 has been demonstrated in mouse lung cells exposed to cigarette smoke. In view of these data, we wanted to investigate whether Egr-1 can be implicated also in the oxysterol metabolism during brain aging. Our results show that Egr-1 expression is differently expressed in the cortex and hippocampus of old mice, as well as the oxysterol profile between these two brain areas. In particular, we show that the cortex experiences in an age-dependent fashion increasing levels of the Egr-1 protein, and that these correlate with the level of HO-1 expression and oxysterol abundance. Such a situation was not observed in the hippocampus. These results are further strenghtened by our observations made with Egr-1 KO mice, confirming our hypothesis concerning the influence of Egr-1 on oxysterol production and accumulation via regulation of the expression of HO-1 in the cortex, but not the hippocampus, of old mice. It is important to notice that most of the oxysterols involved in this process are those usually stimulated by oxidative stress, which would then represent the triggering factor for this mechanism.

## Introduction

Thanks to the efforts made in the past half century in preventing diseases and improving patient conditions, the world population is becoming older. This has certainly been a conquer for human kind in general, but it also raises the question of whether we have for the future to treat aging as a disease factor. In fact, age is a recognized risk factor for adult-onset neurodegenerative processes, including Alzheimer (AD) and Parkinson diseases (PD) (Reitz et al., 2011; Reeve et al., 2014). How aging affects from the molecular point of view the development of neurodegenerative diseases still remains largely undefined.

Numerous studies have recognized Egr-1 as a gene influenced by the aging process (Yau et al., 1996; Desjardins et al., 1997; Blalock et al., 2003; Marrone et al., 2011). Egr-1 is an immediate early gene coding for a zinc finger transcriptional factor involved in cell differentiation, proliferation and survival. Egr-1 expression, which is almost undetectable in mouse embryo nervous system (McMahon et al., 1990), appears and slowly increases in postnatal rat brains reaching consistent levels by day 17 and from that it mantains baseline expression levels until adulthood (Watson and Milbrandt, 1990; Beckmann and Wilce, 1997). In the nervous system, this gene plays an important role in memory and cognition, which are both affected in advanced aging. Relevant changes in Egr-1 expression are observed in rat brain during cortical and hippocampal synaptic formation (Herms et al., 1994), favoring the hypothesis of an involvement of Egr-1 in the regulation of plastic adaptation. In agreement, mice lacking Egr-1 exhibit defects in durable long term potentiation (LTP) and memory (Jones et al., 2001), and Egr-1 is over-expressed in brains of AD patients and AD mouse models (Bakalash et al., 2011; Hendrickx et al., 2013; Gatta et al., 2014). Moreover, Egr-1 is involved in the phosphorylation of tau protein as shown in experiments of Egr-1 deletion and over-expression (Lu et al., 2011). Finally, Egr-1 is rapidly induced by stress stimuli such as hypoxia, OS, brain injury, ischemic stroke, and neuroinflammation (Beckmann et al., 1997; Yan et al., 2000; Khachigian, 2006). OS plays an etiologic role in brain aging, neurodegenerative and cardiovascular diseases, and cancer (Thanan et al., 2014). In this context, we focused our attention on HO-1, a 32 kDa heat-shock protein considered as a new player in aging and neurodegenerative diseses (Chen et al., 2003; Parfenova et al., 2012). In response to OS in the brain, HO-1 is induced in neuronal and glial cells, the greater HO-1 levels being observed within the astrocyte compartment (Manganaro et al., 1995; Snyder et al., 1998). By converting heme to iron, carbon monoxide and biliverdin (Chen and Regan, 2005), HO-1 protects astrocytes against the heme-mediated oxidative injury. On the other hand, HO-1 can also have neurotoxic effects depending on the balance between the free radical scavenging properties of the produced biliverdin/bilirubin (Doré et al., 1999) and the aggravated intracellular OS resulting from the free iron formation (Zhang and Piantadosi, 1992). HO-1 expression has not been rigourously localized in the human brain. In normal conditions, HO-1 is scanty present in neuroglia and in some neuron sub-populations from various brain areas, including cortex and hippocampus (Vincent et al., 1994; Nakaso et al., 2000).

Since the brain is the organ with the highest content of cholesterol (approximately 20% of total body cholesterol content), the role of sterols homeostasis and in particular cholesterol metabolism, is of primary importance in the study of aging related disorders. An interaction of HO-1 induction with sterol regulation was reported both in *in vitro* and *in vivo* studies (Vaya et al., 2007; Hascalovici et al., 2009, 2014). The aged and neurodegenerated brain is constantly exposed to OS, which leads to lipid peroxidation and to the generation of oxysterols (Björkhem et al., 2002), oxygenated forms of cholesterol arising also from OS (Iuliano, 2011). Oxysterols are able to induce cytotoxicity and inflammation, worsening the neurodegeneration process (Vaya and Schipper, 2007). Certain oxysterols are used to control brain cholesterol levels, which is synthesized *in situ* and unable to cross the blood brain barrier. A net flux of 24-HC from the brain to the systemic circulation serves to export cholesterol from the brain (Crick et al., 2015; Iuliano et al., 2015).

To prevent accumulation in the brain, cholesterol is oxidized by the cytochrome p450 enzymes. During aging, it is the balanced production of cholesterol and oxysterols to provide the basis for oxysterol homeostasis in the brain. When this equilibrium is perturbated, the accumulation of oxysterols in the brain can be toxic. In AD patients the level of inflammatory molecules and the expression of HO-1 were related to changes in oxysterols profile and disease progression (Testa et al., 2016). A link between Egr-1 and HO-1 was reported in mouse lung cells exposed to cigarettes smoke (Chen et al., 2010). With this work we have advanced the hypothesis of a contribution of Egr-1 in the regulation of oxysterol production in the aging brain. To test our hypothesis, we studied the expression of Egr-1 and HO-1 in the cortex and hippocampus of young, adult and aged WT and Egr-1 KO mice, and measured the amount of oxysterols at tissue level. Our results are consistent with the following outcome: Egr-1 regulates oxysterols production by controlling the expression of HO-1. This association is clearly present in the brain cortex but not in the hippocampus, and it seems operative in an age-dependent manner.

## Materials and Methods

### Animals

Male C57BL/6 WT and Egr-1 KO mice aged 3, 12, and 24 months were used (*n* = 6 animals per strain and age). Animals were housed 3 to 4 per cage, maintained under standard temperature conditions (22°C ± 2°C) and 12-h:12-h light:dark cycles. Throughout the study, they had access to food and water *ad libitum*. All the experiments were performed in accordance with the EU Directive 2010/63/EU for animal experiments and approved by ethic committee of Department of Medico-Surgical Sciences and Biotechnologies, University of Roma “Sapienza”.

### Brains Processing

For Western blot analysis and oxysterols determinations, mice were sacrificed by cervical dislocation and the brains were immediately removed and washed in ice cold Phosphate Buffer Saline (PBS). Afterward, brains were divided into two emispheres practicing a sagittal cut and both brain cortices and hippocampi were accurately dissected, frozen on powdered dry ice, and maintained at -80°C until protein extraction and oxysterols analysis.

For immunohistochemistry analysis, the whole brain was extracted, washed in ice cold PBS and fixed for 24h at 4°C with Carnoy’s solution. Afterward, brains were mantained in 70% ethanol untill the paraffin-embending process.

### Immunohistochemistry

Immunohistochemical analysis was conducted as previously described by our research group (Rosa et al., 2017), with some modifications. In brief, paraffin-embedded tissues were deparaffinized, rehydrated in descending graded alcohols, incubated for 15 min in methanol containing 3% H_2_O_2_ to block endogenous peroxidase activity, and then subjected to microwave antigen retrieval for 30 min in sodium citrate buffer (10 mM tri-sodium citrate dihydrate, 0.05% Tween-20, pH 6.0). After preincubation in Super Block (ScyTek Laboratories, Logan, UT, United States) for 10 min, sections were incubated overnight with rabbit polyclonal anti-Egr-1 antibody (sc-110, Santa Cruz Biotechnology, Dallas, TX, United States, dilution 1:100) or rabbit polyclonal anti- HO-1 antibody (HO-1, sc-10789, Santa Cruz Biotechnology, dilution 1:100) at 4°C in humid chamber, washed with PBS, incubated for 10 min at room temperature with UltraTek Anti-Polyvalent (ScyTek Laboratories), washed with PBS and then incubated with the UltraTek HRP (ScyTek Laboratories) according to the manufacturer’s instructions. The sections were then stained with 3-3-diaminobenzidine (ScyTek Laboratories) as chromogen to visualize the reaction product, and were finally counterstained with hematoxylin. Images were acquired with Nikon Eclipse Ni motorized microscope system at 10× and 40× magnification.

### Protein Extraction

Total protein extraction was performed homogenizing cortical and hippocampal samples with a Teflon-glass potter in RIPA buffer (50 mM Tris–HCl pH 8.0, 150 mM NaCl, 1% Nonidet P-40, 1 mM EDTA, 0.5% sodium deoxycholate, 0.1% SDS) with protease inhibitors, 1 mM PMSF, 1mM DTT, and 0.5 mM sodium orthovanadate (Sigma–Aldrich, St. Louis, MO, United States). Protein concentration was determined by the Bradford assay (Bio-Rad, Hercules, CA, United States).

### Western Blot

Western blot analysis of brain extracts was carried out as previously described (Ponti et al., 2015), with some modifications. In brief, 40 μg of proteins per sample were resolved on a 10% SDS–PAGE gel and blotted onto a PVDF membrane (Amersham HyBond-P GE Healthcare, Chicago, IL, United States). After blocking at room temperature in 5% dry-milk in PBS containing 0.1% Tween-20 for 1 h, membranes were incubated overnight at 4°C with rabbit polyclonal anti-heme oxygenase 1 (HO-1, sc-10789, Santa Cruz Biotechnology, dilution 1:500), rabbit polyclonal anti-Egr-1 (sc-110, Santa Cruz Biotechnology, dilution 1:500), rabbit polyclonal anti-ERK1/2 (9102, Cell Signaling Technology, Danvers, MA, United States dilution 1:1000), mouse monoclonal anti-phospho-ERK1/2 (p-ERK1/2, 9106, Cell Signaling Technology, dilution 1:1000) and mouse monoclonal anti-β-actin (sc-47778, Santa Cruz Biotechnology, dilution 1:2000) antibody was used for normalization. Membranes were then incubated with anti-rabbit and anti-mouse horseradish peroxidase conjugated secondary antibodies (dilution 1:10000, GE Healthcare Bio-Sciences, Piscataway, NJ, United States). Immunocomplexes were detected by ECL Western Blotting detection system (GE Healthcare Bio-Sciences). Digital images were acquired using a ChemiDoc XRS + System (BioRad). Band intensities were quantified by densitometric analysis using Image Lab software (BioRad), and values were normalized to β-actin.

### Oxysterols and Cholesterol Quantification

Cortical and hippocampal samples from 12 to 18 months old WT and Egr-1 KO mice (*n* = 6 for each group) were used for oxysterols determinations. In brief, oxysterols were measured by isotope dilution mass spectrometry, essentially as described elsewhere (Iuliano et al., 2003) with the exception of the solid phase extraction (SPE) step, which was repeated twice to eliminate cholesterol. Cholesterol discarded by elution from SPE was measured by isotope dilution mass spectrometry such as oxysterols. Analyses were run on an Agilent 6890N gas chromatograph equipped with a 7683 series automatic liquid sampler, and interfaced with an Agilent 5973 mass spectrometer (Agilent Technologies, Palo Alto, CA, United States). Separation was achieved on a 30 m capillary column (HP-5MS 30 0.25 mm ID, 0.25 mm thickness). The mass spectrometer was set to the selected ion monitoring mode; the molecules were monitored with ions at mass/charge ratio (m/z): 463 m/z for [^2^H_7_]-27-HC, [^2^H_7_]-24-HC, [^2^H_7_]-7α-HC, [^2^H_7_]-7β-HC, [^2^H_7_]-4α-HC, [^2^H_7_]-4β-HC and [^2^H_7_]-cholesterol; 456 m/z for [^2^H_0_]-27-HC, [^2^H_0_]-24-HC, [^2^H_0_]- 7α-HC, [^2^H_0_]-7β-HC, [^2^H_0_]-4α- hydroxycholesterol (4α-HC), [^2^H_0_]-4β-HC and [^2^H_0_]-cholesterol; 481 m/z for [^2^H_7_]-5α,6α-EC, and [^2^H_7_]-5β,6β-EC; 474 m/z for [^2^H_0_]-5α,6α-EC, and [^2^H_0_]-5β,6β-EC; 137 m/z for [^2^H_6_]-25-HC, and 131 m/ z for [^2^H_0_]-25-HC; 479 m/z for [^2^H_7_]-7-KC, and 472 m/z for [^2^H_0_]-7-KC; 478 m/z for [^2^H_6_]-5α-OH,6-oxocholesterol and 472 m/z for [^2^H_0_]-5α-OH,6-oxocholesterol (5α-OH,6-oxo-CH); 546 m/z for [^2^H_7_]-CT, and 552 m/ z for [^2^H_0_]-CT; 189 m/z for [^2^H_7_]- 22-HC and 181 m/z for [^2^H_0_]- 22-HC. 5α-OH,6-oxo-CH, CT and theirs deuterated were synthesized and kindly donated by Dr. Marc Poirot (Institut Claudius Régaud, Toulouse, France). 4α-HC, 4β-HC and theirs deuterated were kindly donated by Dr. G. Lizard (Faculté des Sciences Gabriel, Dijon, France). All other oxysterols and deuterated oxysterols were from Avanti Polar Lipids (Alabaster, AL, United States). Quantification of oxysterols was assessed by the internal standard ratio method.

### Statistical Analysis

All statistical analyses were performed using GraphPad Prism software (La Jolla, CA, United States). Western blot results are expressed as the mean ± standard deviation (SD) and statistical comparisons were performed by *Student’s t-*test and *one-way analysis of variance* (ANOVA). *P*-values < 0.05 were considered significant.

## Results

### Egr-1 and Heme Oxygenase-1 Have a Different Distribution in the Brain Cortex and Hippocampus of Adult Wild-Type Mice

**FIGURE 1:**
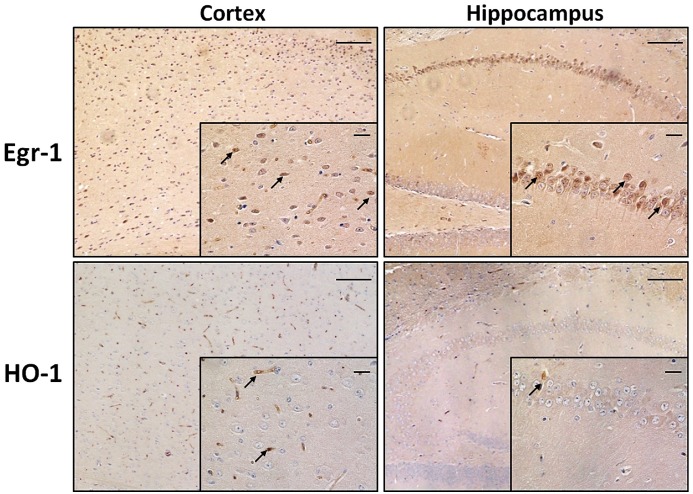
Early growth response-1 and HO-1 expression in adult wild-type mice cortex and hippocampus. Immunohistochemical analysis showing representative brain sections from cortex (left images) and hippocampus (right images) of adult wild-type (WT) mice stained with anti-Egr-1 (upper images) and anti-HO-1 (bottom images) antibodies. Black arrows indicate strongly positive signals. Images: 10× magnification, scale bars: 100 μm; inside squares: 40× magnification, scale bars: 20 μm.

Among the brain areas involved in memory and cognition, cortex and hippocampus are exposed to morphological and functional changes as age advances. First of all we wanted to compare the pattern of expression of Egr-1 and HO-1 in cortex and hippocampus of adult WT mice. They were stained by immunohistochemistry on paraffin-embedded sections. Figure 1 shows Egr-1 in the brain cortex with its typical nuclear localization, and in the hippocampus within the pyramidal neurons of CA (CA1-CA2-CA3-CA4) but not in the DG. On the other hand, a weak presence of HO-1 was detected in the cortex and hippocampus areas, mainly around vessels.

### Oxysterols Have Different Profiles in the Brain Cortex and Hippocampus of Wild-Type Mice

**FIGURE 2:**
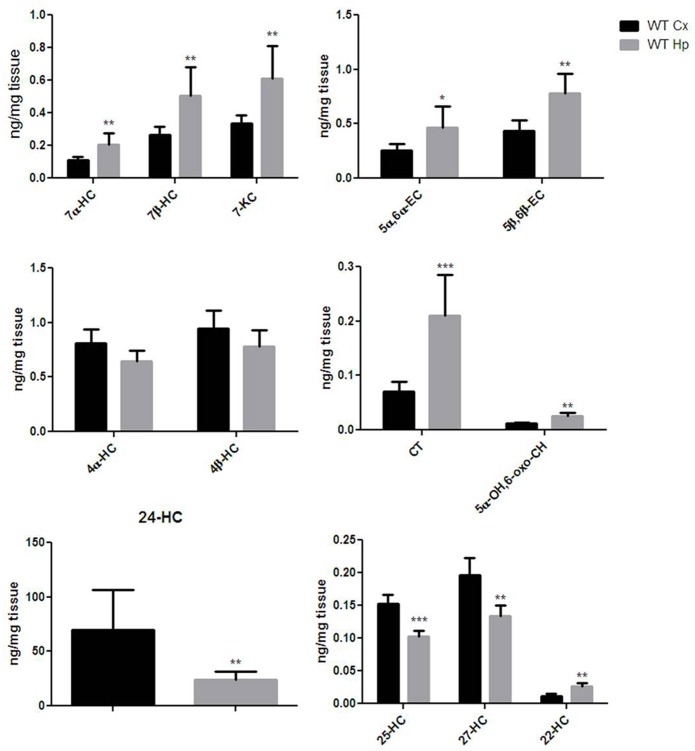
Different oxysterols profile between adult wild-type mice cortex and hippocampus. Isotope dilution mass spectrometry analysis showing oxysterols levels in 12 months WT mice cortex (Cx, black bars) and hippocampus (Hp, gray bars) expressed as ng/mg of tissue. 7α-hydroxycholesterol (7α-HC), 7β-hydroxycholesterol (7β-HC), 7-ketocholesterol (7-KC), 5α,6α-epoxycholesterol (5α,6α-EC), 5β,6β-epoxycholesterol (5β,6β-EC), 4α-hydroxycholesterol (4α-HC), 4β-hydroxycholesterol (4β-HC), 5α-cholestane-3β,5,6β-triol (CT), 5α-OH,6-5α-OH,6-oxocholesterol (5α-OH,6-oxo-CH), 24-hydroxycholesterol (24-HC), 25-hydroxycholesterol (25-HC), 27-hydroxycholesterol (27-HC), 22-hydroxycholesterol (22-HC). Results are presented as the mean ± standard deviation (*n* = 6). ^∗^*p* < 0.05; ^∗∗^*p* < 0.01; ^∗∗∗^*p* < 0.001.

Extensive OS and oxysterol production are emerging components of the neurodegenerative processes involved in brain aging (Sottero et al., 2009). In order to get an oxysterol profile for the cortex and hippocampus of adult WT mice, we investigated the production of 13 different oxysterols. As shown in Figure 2, the oxysterols included in our panel were five of non-enzyimatic origin (7-KC, 7β-HC, 5α-OH,6-oxo-CH, 5α,6α-EC, and 5β,6β-EC), five of enzimatic origin (4β-HC, 4α-HC, 24-HC, 27-HC, and 22-HC), and three of both enzymatic and non-enzymatic origin (25-HC, CT and 7α-HC). The cortex and hippocampus showed distinct oxysterol profiles. The hippocampus was characterized by higher levels of oxysterol 7α-HC, 7β-HC, 7-KC, 5α,6α-EC, 5β,6β-EC, CT, 5α-OH,6-oxo-CH and 22-HC, whereas the cortex had higher levels of 24-HC, 25-HC and 27-HC. 4α-HC and 4β-HC were similarly distributed between cortex and hippocampus. Cholesterol levels (Supplementary Figure S1) measured in the same regions did not result in significant difference in the two brain areas of both WT and KO mice, either at 12 and 18 months of age.

### Egr-1 Protein Expression Increases in the Cortex but Not in the Hippocampus During Aging of Wild-Type Mice

**FIGURE 3:**
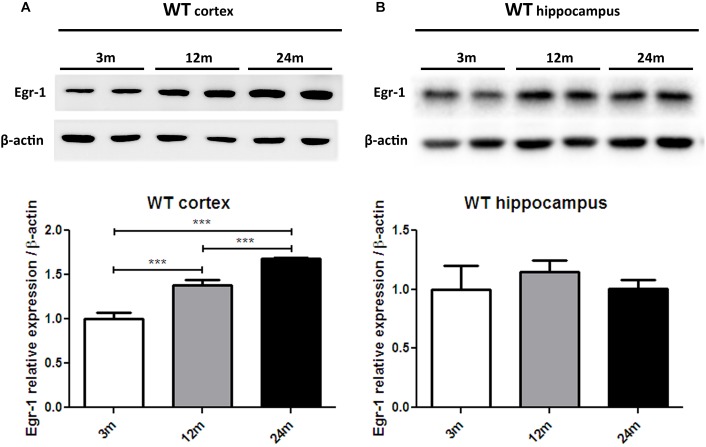
Egr-1 protein expression in the cortex and hippocampus of young, adult and aged wild-type mice. Protein levels of Egr-1 in the cortex **(A)** and hippocampus **(B)** of 3, 12 and 24 months old WT mice. The image shows representative western blots (upper panels) and bar graphs (bottom panels) obtained from the relative densitometric analysis, as described in Materials and Methods. Results are presented as the mean ± standard deviation (*n* = 6). ^∗∗∗^*p* < 0.001.

In order to advance the hypothesis of whether Egr-1 is involved in oxysterol metabolism changes in brain aging, we checked the levels of Egr-1 protein expression by western blot in the brain cortex (Figure 3A) and hippocampus (Figure 3B) of young (3 months), adult (12 months) and aged (24 months) WT mice. Egr-1 levels were found to increase 1.4 folds from three to 12 months and 1.8 folds up to 24 months. These changes were observed only in the cortex, suggesting a possible role of Egr-1 for cortex aging.

### Egr-1 Is Sustained by ERK1/2 Phosphorylation and Is Likely Involved in the Heme Oxygenase-1 Regulation in the Brain Cortex of Aged Wild-Type Mice, but Not in the Hippocampus

**FIGURE 4:**
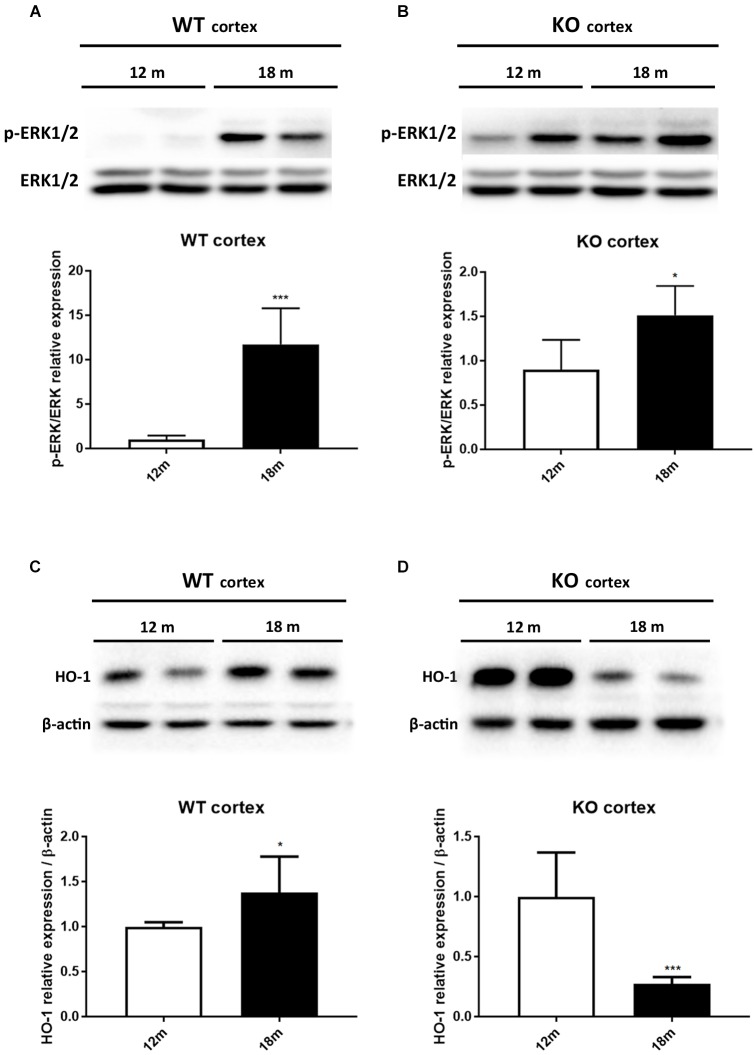
ERK1/2 phosphorylation and HO-1 protein expression in the cortex of adult and aged wild-type and Egr-1 KO mice. ERK1/2, p-ERK1/2 and HO-1protein levels in the cortex of 12 and 18 months old WT **(A–C)** and Egr-1 KO **(B–D)** mice. The image shows representative western blots (upper panels) and bar graphs (bottom panels) obtained from the relative densitometric analysis, as described in Materials and Methods. Results are presented as the mean ± standard deviation (*n* = 6). ^∗^*p* < 0.05; ^∗∗∗^*p* < 0.001.

**FIGURE 5:**
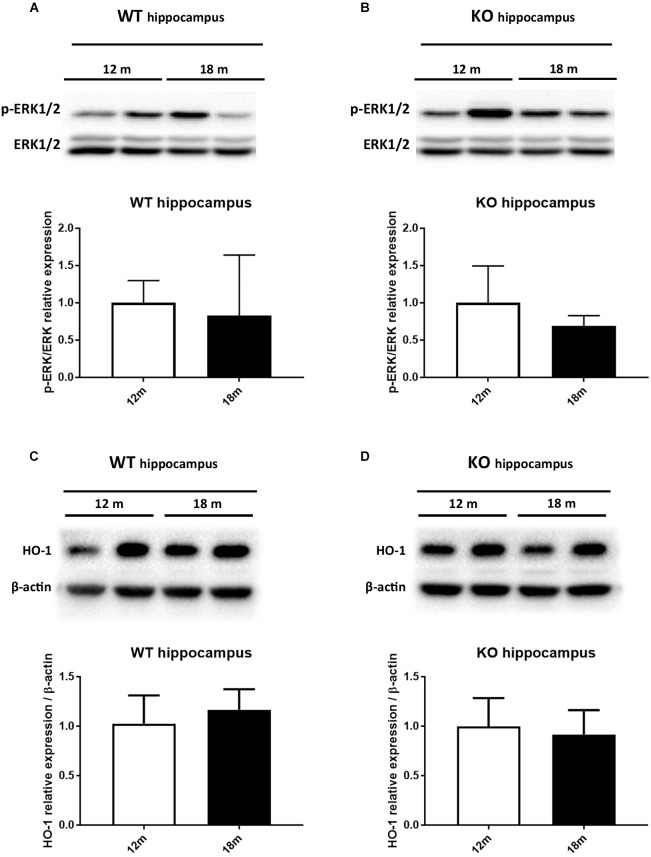
ERK1/2 phosphorylation and HO-1 protein expression in the hippocampus of adult and aged wild-type and Egr-1 KO mice. ERK1/2, p-ERK1/2 and HO-1 protein levels in the hippocampus of 12 and 18 months old WT **(A–C)** and Egr-1 KO **(B–D)** mice. The image shows representative western blots (upper panels) and bar graphs (bottom panels) obtained from the relative densitometric analysis, as described in Materials and Methods. Results are presented as the mean ± standard deviation (*n* = 6).

It has been reported that HO-1 is involved in oxysterol metabolism during neurodegeneration (Leoni, 2009; Schipper et al., 2009). It has been also shown that Egr-1 regulates HO-1 expression induced by cigarette smoke in mouse lung cells (Chen et al., 2010). Furthermore, the Egr-1 promoter has been described to be activated by the extracellular signal-regulated kinase (ERK) 1/2, which has been documented to be phosphorylated by many stress stimuli, including OS (Park and Koh, 1999; Hartney et al., 2011; Chen et al., 2012). Based on these observations, we compared by western blot the levels of ERK1/2 phosphorylation (Figures 4A,B) and HO-1 (Figures 4C,D) expression in the cortex of adult (12 months) and aged (18 months) WT (Figures 4A–C) and Egr-1 KO (Figures 4B–D) mice. Our results show that in WT mice the phosphorylation of ERK1/2 and HO-1 expression are the highest in the cortex of 18 months old mice, where also the Egr-1 levels are highest. In contrast, HO-1 expression is significantly reduced in the cortex of age-matched Egr-1 KO mice, although the stronger phosphorylation of ERK1/2. Similar experiments to assay in the hippocampus the link between Egr-1 and HO-1 were performed. No statistically significant differences between the levels of ERK1/2 phosphorylation (Figures 5A,B) and the expression of HO-1 (Figures 5C,D) in the hippocampus of adult and aged WT (Figures 5A–C) and Egr-1 KO mice (Figures 5B–D) were observed.

### Egr-1 Expression Is Pivotal to the Oxysterol Production in the Brain Cortex of Aged Wild-Type Mice

**FIGURE 6:**
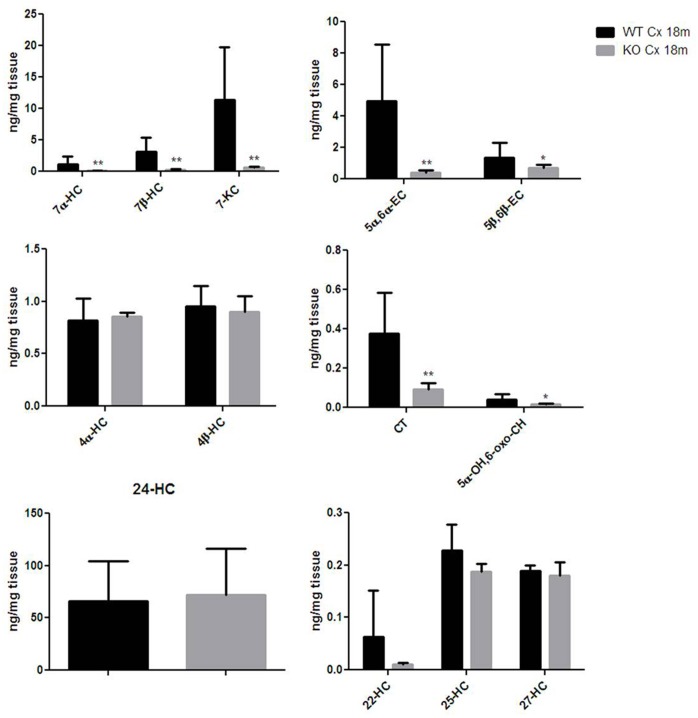
Oxysterols levels in aged wild-type and Egr-1 KO mice brain cortex. Isotope dilution mass spectrometry analysis showing oxysterols levels in 18 months wild-type (WT, black bars) and Egr-1 knock-out (KO, gray bars) mice cortex (Cx) expressed as ng/mg of tissue. 7α-HC, 7β-HC, 7-KC, 5α,6α-EC, 5β,6β-EC, 4α-HC, 4β-HC, CT, 5α-OH,6-oxo-CH, 24-HC, 25-HC, 27-HC, 22-HC. Results are presented as the mean ± standard deviation (*n* = 6). ^∗^*p* < 0.05; ^∗∗^*p* < 0.01; ^∗∗∗^*p* < 0.001.

Thereafter, we were asking whether the correlation found between the Egr-1 and HO-1 expression levels in the cortex of aging mice were reflected also in a change of oxysterol levels. We then compared the oxysterol levels in the old WT brain cortex with those found in old Egr-1 KO mice brain cortex. Figure 6 shows that in the cortex of old KO mice the oxysterols 7α-HC, 7β-HC; 7-KC, 5α,6α-EC, 5β,6β-EC, CT and 5α-OH,6-oxo-CH were significantly reduced.

### Hippocampal Expression of Egr-1 Is Not Involved in Oxysterols Metabolism

**FIGURE 7:**
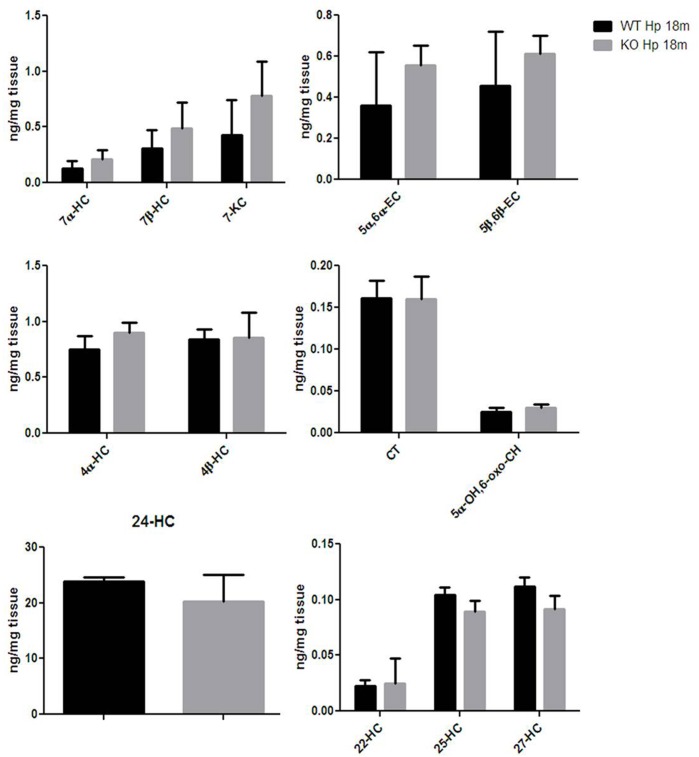
Oxysterols levels in aged wild-type and Egr-1 KO mice hippocampus. Isotope dilution mass spectrometry analysis showing oxysterols levels in 18 months wild-type (WT, black bars) and Egr-1 knock-out (KO, gray bars) mice hippocampus (Hp) expressed as ng/mg of tissue. 7α-HC, 7β-HC, 7-KC, 5α,6α-EC, 5β,6β-EC, 4α-HC, 4β-HC, CT, 5α-OH,6-oxo-CH, 24-HC, 25-HC, 27-HC, 22-hydroxycholesterol (22-HC). Results are presented as the mean ± standard deviation (*n* = 6).

Finally, given that HO-1 expression does not seem, according to our data, to be influenced by Egr-1 in the hippocampus, we asked the question of whether also oxysterol metabolism appeared to gain independence from Egr-1 control in old mice hippocampus. As shown in Figure 7, we haven’t registered any significative change in the content of oxysterols in the hippocampus of aged mice, though 7α-HC, 7β-HC, 7-KC, 5α,6α-EC and 5β,6β-EC showed an upward trend.

## Discussion

In this study about brain aging, we brought to light a new regulatory circuit linking the early transcriptional factor Egr-1 to the brain oxysterol production and accumulation in the elderly. Moreover, Egr-1, likely by regulating the expression of HO-1, could have an influence in the accumulation of oxysterols in the aged brain, specifically in the cortex. The results reported here show the increase in Egr-1, the 32 kDa heat shock protein HO-1 and oxysterol levels in an age-related manner in the mouse cortex. These changes do not occur in the hippocampus area, nor are they observed in the cortex of age-matched Egr-1 KO mice, highlighting the likely role of Egr-1 in this process. Egr-1 is a stress-induced early gene and a transcriptional factor able to regulate the expression of several genes involved in cell survival, death and proliferation. Several studies have established an involvement of Egr-1 in synaptic plasticity and cognitive function, but completely unknown is whether Egr-1 is involved in their natural decline with age as well. On the other hand, Egr-1 is implicated in the development of neurodegenerative disorders, thus becoming a hallmark for such diseases (Koldamova et al., 2014; Minatohara et al., 2015; Duclot and Kabbaj, 2017). In AD brains, Egr-1 is found to be upregulated (Gómez Ravetti et al., 2010), while in aged mice lacking Egr-1 LTP is impaired (Jones et al., 2001). The aging brain is constantly exposed to OS, which triggers and exacerbates brain functional deficit (Uttara et al., 2009). Oxysterols as a form of oxidized cholesterol are implicated in OS, in particular the B-ring oxysterols are generated by autoxidation and by free radical-mediated mechanisms (Iuliano, 2011) and are regulated by HO-1, an enzyme with a dual function that can be protective or toxic depending on its levels and context (Chen, 2014).

Based on the above assumptions about the role of Egr-1 in brain, we wanted to investigate the expression of Egr-1, HO-1 and the levels of oxysterols in the cortex and hippocampus of adult and old mice, i.e., in an age-dependent fashion, to establish whether a link could exist between the levels of Egr-1 and OH-1, and between the level of Egr-1 and the type and amount of oxysterol accumulated, so to infer about and what consequences could a change in these levels of Egr-1 and of HO-1 may have on the accumulation of oxysterols in the same areas. To the purpose of challenging the effect of Egr-1 on the expression of HO-1 and oxysterol accumulation, we gathered data from WT and Egr-1 KO mice and compared the results according to the area of interest (cortex or hippocampus), and the mouse age (12 or 18 months).

Our data highlight some differences in the regulation of Egr-1 protein expression in the mouse brain cortex and hippocampus in the aging brain from WT mice. We found that Egr-1 expression increases in the cortex in an age-dependent fashion. We can only speculate about the cause behind our evidences. They can be likely due to a response to the many stress stimuli, including OS, hypoxia and inflammation that occur in the aged brain. In the hippocampus, however, Egr-1 does not seem to be under the same kind of regulation. In fact, we did not observe any significant change of expression. These data are in agreement with a previous study showing that Egr-1 levels in the brain do not change with age in resting mice, but only when they are exposed to a learning task (Desjardins et al., 1997).

Brain oxysterols are widely accepted markers of OS (Leoni and Caccia, 2011). Several studies have focused on 24-HC and 27-HC, which are produced by enzymatic routes and play an important role in neurodegenerative diseases, Alzheimer’s included (Bjorkhem et al., 2006; Hughes et al., 2013). For the first time to our knowledge, we provided a comprehensive oxysterol profile for the cortex and hippocampus of adult mice. We showed that distribution and abundance of oxysterols was different between cortex and hippocampus, and highlighted that two distinct brain areas, with different functions, have their own oxysterol composition.

Heme oxygenase-1 is considered a key enzyme for oxysterol metabolism (Vaya et al., 2007; Hascalovici et al., 2009). In this context, old mice cortex has increased levels of HO-1 compared to younger mice. This result is in agreement with other studies reporting that HO-1 increases with age, is induced by stress and the high levels correlate with cognitive deficits (Schipper, 1999, 2004; Hirose et al., 2003). Surprisingly, in absence of Egr-1 the expression of HO-1 in the cortex was strongly inhibited in old mice compared to younger KO mice. This result is in line with previous observations, although in mouse lung cells exposed to cigarette smoke, where Egr-1 was able to regulate HO-1 (Chen et al., 2010). Finally, our data showed that this signaling may be sustained by the phosphorylation of ERK1/2 in the old brain cortex, which has already been described to be more activated during aging and to be associated to synaptic dysfunction (Ogundele et al., 2018). Furthermore, in smooth muscle cells Egr-1 was demonstrated to be activated via ERK1/2 under OS conditions (Pagel and Deindl, 2012).

**FIGURE 8:**
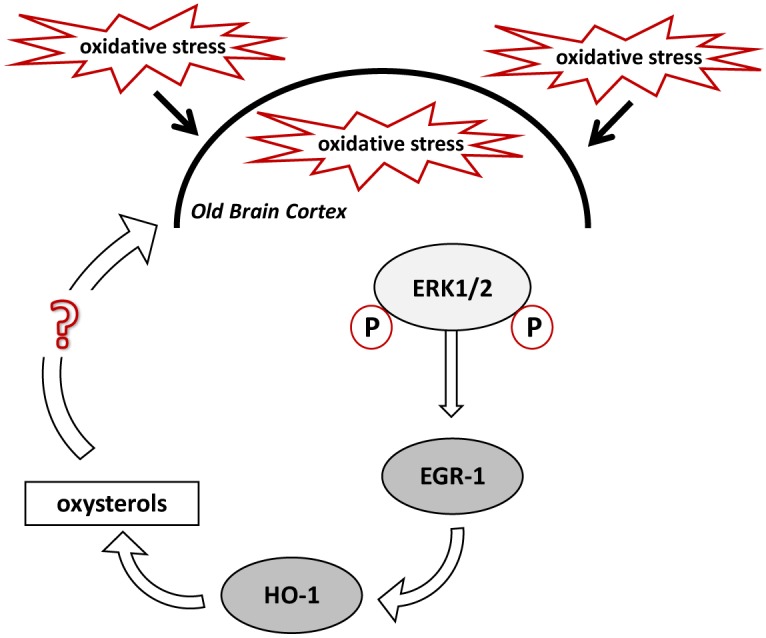
Proposed mechanism for oxysterols production in the old mice brain cortex. High levels of oxidative stress (OS) experienced by the aged mouse brain cause the phosphorylation of ERK1/2, which induce EGR-1 in the cortex. EGR-1, in turn, activates HO-1, which regulates oxysterols metabolism. Specific oxysterols, or a group of them, may maintain and exacerbate the OS within the brain cortex.

Based on our hypothesis, stimulation of HO-1 in the brain cortex of old mice should result in enhanced oxysterol levels, whereas deletion of Egr-1 should interfere with this process. In fact, old WT mice cortex showed higher levels of oxysterols than the cortex of younger mice but not in the old Egr-1 KO mice which presented much lower oxysterol levels. It is important to notice that the changes concern mainly those oxysterols which have been demonstrated to be induced by OS, i.e., 7α-HC, 7β-HC, 7-KC, 5α,6α-EC and 5β,6β-EC (Iuliano, 2011; Mutemberezi et al., 2016). We suggest that oxysterol production in the aging brain cortex is based on the oxidative induction of Egr-1 via ERK1/2 phosphorylation, which, in turn, activates HO-1 (Figure 8). It would be interesting to explore whether a single or a group of oxysterols may act themselves as a source of OS able to maintain ERK1/2 in a phosphorylated state and Egr-1 levels high.

Since we have reported the absence of stimulation of Egr-1 and no difference in ERK1/2 phosphorylation in the hippocampus of old mice, we finally wanted to examine whether HO-1 and oxysterols expression was in this case not anymore driven by an Egr-1-dependent regulation in this brain area. Our results confirmed that the HO-1 and oxysterol levels were not significantly different between the hippocampus of adult and of old WT and Egr-1 KO mice, confirming our hypothesis.

In conclusion, our results show that Egr-1 can be stimulated in the cortex of old mice suggesting a role in the oxysterol accumulation by regulating the HO-1 expression. This new function covered by Egr-1 seems to be brain region-specific because it is not present in the hippocampus.

## Author Contributions

PR conceived and designed the experiments. PR, CZ, and ACr performed the experiments. PR, CZ, A-MC, LI, and GR analyzed the data. ACa, ACo, and LI contributed reagents, materials, and analysis tools. PR, ACo, A-MC, and ACa wrote the paper. LI and GR revised the paper.

## Conflict of Interest Statement

The authors declare that the research was conducted in the absence of any commercial or financial relationships that could be construed as a potential conflict of interest.

## Supplementary Material

FIGURE S1 Cholesterol levels in adult and aged wild-type and Egr-1 KO mice brain cortex and hippocampus. Isotope dilution mass spectrometry analysis showing cholesterol levels in 12 (white bars) and 18 months (black bars) WT and Egr-1 knock-out (KO) mice cortex (Cx) and hippocampus (Hp) expressed as μg/mg of tissue. Results are presented as the mean ± standard deviation (*n* = 6).

## Abbreviations

22-HC: 22-hydroxycholesterol
24-HC: 24-hydroxycholesterol
25-HC: 25-hydroxycholesterol
27-HC: 27-hydroxycholesterol
4α-HC: 4α-hydroxycholesterol
4β-HC: 4β-hydroxycholesterol
5α,6α-EC: 5α,6α-epoxycholesterol
5β,6β-EC: 5β,6β-epoxycholesterol
5α-OH,6-oxo-CH: 5α-OH,6-5α-OH,6-oxocholesterol
7α-HC: 7α-hydroxycholesterol
7β-HC: 7β-hydroxycholesterol
7-KC: 7-ketocholesterol
CA: Cornu Ammonis
CT: 5α-cholestane-3β,5,6β-triol
Cx: cortex
DG: Dentate Girus
Egr-1: Early growth response-1
HO-1: heme oxygenase-1
Hp: hippocampus
KO: knock-out
OS: oxidative stress
WT: wild-type
