# Supplementary figures and images for: Heme Oxygenase-1 and Brain Oxysterols Metabolism Are Linked to Egr-1 Expression in Aged Mice Cortex, but Not in Hippocampus
Source: Front Aging Neurosci. 2018 Nov 6;10:363. doi: 10.3389/fnagi.2018.00363 (PMC6232516; doi:10.3389/fnagi.2018.00363)

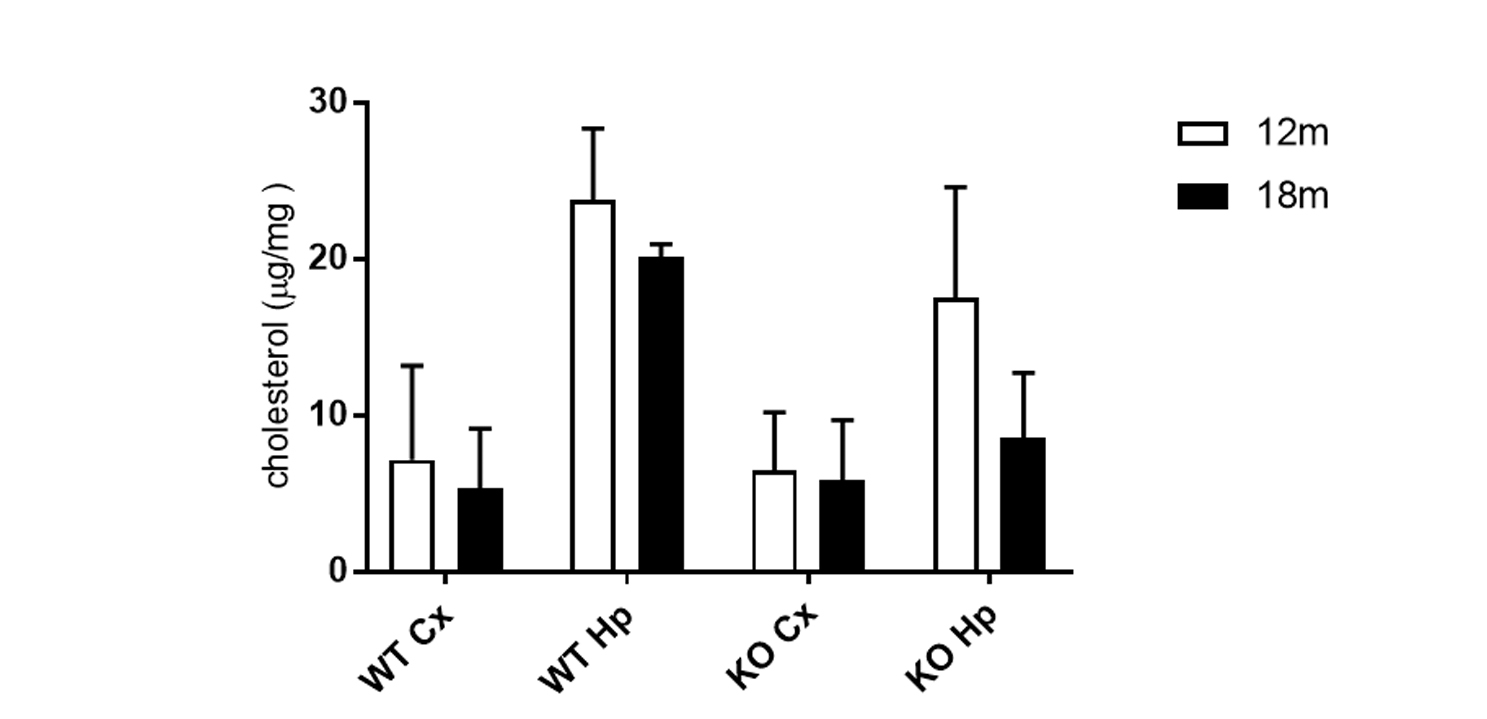

Supplement: FIGURE S1 — Cholesterol levels in adult and aged wild-type and Egr-1 KO mice brain cortex and hippocampus. Isotope dilution mass spectrometry analysis showing cholesterol levels in 12 (white bars) and 18 months (black bars) WT and Egr-1 knock-out (KO) mice cortex (Cx) and hippocampus (Hp) expressed as μg/mg of tissue. Results are presented as the mean ± standard deviation (n = 6). [file Image_1.JPEG]
